# Inpatient continuous glucose monitoring halves the risk of hypoglycemia in patients with hyperinsulinism

**DOI:** 10.3389/fendo.2026.1943475

**Published:** 2026-10-15

**Authors:** Chloe Chandra, Shamani De Silva, Sarah Worthington, Indraneel Banerjee, Chris Worth

**Affiliations:** 1 Faculty of Biology, Medicine and Health, University of Manchester, Manchester, United Kingdom; 2 Royal Manchester Children’s Hospital, Department of Pediatric Endocrinology, Manchester University NHS Foundation Trust, Manchester, United Kingdom

**Keywords:** congenital hyperinsulinism, continuous glucose monitoring, hypoglycemia, inpatient monitoring, pediatric endocrinology

## Abstract

**Introduction:**

Patients with congenital hyperinsulinism (HI) face recurrent and unpredictable hypoglycemia. Conventional inpatient monitoring relies on intermittent fingerprick glucose testing, which is burdensome for pediatric patients and risks missing hypoglycemic episodes between tests.

Continuous glucose monitoring (CGM) provides high granularity, complimentary monitoring but with unproven efficacy to detect and reduce hypoglycemia in the inpatient setting. This study evaluates whether CGM can improve inpatient safety through the early detection, and subsequent treatment, of hypoglycemic episodes.

**Methods:**

Thirteen inpatients with HI were monitored using Dexcom G7 or G6 device. Finger-prick glucose monitoring was performed at the expert clinician’s discretion and to verify CGM-prompted hypoglycemia alerts. All CGM data were retrospectively searched for hypoglycemia episodes. Fingerprick glucose data were concurrently analysed, alongside electronic health records, to quantify early hypoglycemia detection and subsequent hypoglycemia saved. CGM accuracy was analysed using established metrics, including hypoglycemia sensitivity and error grid analysis, in Python 3.1.1.

**Results:**

From 3504 paired CGM and fingerprick values, hypoglycemia sensitivity was 56.7%, with CGM devices tending to overread, by a mean of +0.43mmol/L. On Hypoglycemia Error Grid analysis, 6.2% of values were classed as moderate or severe risk.

Patient data, from 627 days of monitoring, showed 721 CGM-detected hypoglycemia events (<3.5 mmol/L). Of these, 448 (62.1%) were refuted as false positives and 273 (37.9%) were confirmed as true hypoglycemia, by fingerprick glucose monitoring. Patients spent a mean of 16 minutes/day (1.1% time) in hypoglycemia. CGM alerts saved an estimated mean of 21 minutes/day of hypoglycemia; a more than halving of total hypoglycemia experienced per patient.

**Discussion:**

The use of CGM within the inpatient setting, for those with HI, allows for a significant reduction in hypoglycemia; potentially halving hypoglycemic time. Sensor accuracy remains poor in the inpatient setting, resulting in high false negatives and suboptimal hypoglycemia sensitivity.

However, the overall burden of CGM use is relatively low in the inpatient setting, when healthcare professionals can share the work with parents.

## 1 Introduction

Congenital hyperinsulinism (HI) is a rare disorder of glucose homeostasis that usually presents in the neonatal period. It is a heterogenous condition, with the unifying feature of excessive and unregulated insulin secretion resulting in recurrent and severe hypoglycemia (1) and an associated, high risk of neurodevelopmental injury (2–4). The majority of severe cases have an underlying genetic cause, most often mutations in ABCC8 or KCNJ11, resulting in defects in the ATP sensitive K channel (5) and an uncoupling of glucose levels and insulin secretion.

Despite recent progress (6) medical and surgical therapies for HI remain limited (7) and prevention of hypoglycemic brain injury is dependent on regular monitoring of glucose levels and timely adaptation of treatment. In the inpatient setting, patients often have severe disease, necessitating high-frequency blood sampling and frequent alterations in infusions of dextrose-containing intravenous glucagon. Glucose monitoring is invariably performed by intermittent fingerprick glucose sampling but this risks missing hypoglycemia between tests and offers no information on glucose trends (8).

After almost 20 years of use in people living with diabetes, CGM is now being used in the HI population (8, 9) and offers an attractive alternative to intermittent fingerprick glucose monitoring. Despite suboptimal accuracy in the outpatient setting (9, 10) and some negative reports around alarms and sensor changes (11), most HI families report positive feelings around CGM (10–12) and there have been recent calls from HI charity groups to expand its use (13).

Although no large-scale trials have evaluated the efficacy of CGM in the outpatient setting, small studies indicate possible utility as a behavior change tool to reduce hypoglycemia (14, 15). Furthermore, use of inpatient CGM data has been used to provide high-granularity phenotyping data (16, 17) and report on clinical trial outcomes (6). CGM as an inpatient tool has multiple theoretical benefits such as minimizing missed hypoglycemia between tests; timely titration of intravenous therapies in response to glucose levels; reducing painful finger-prick glucose tests; possible diagnostic use (9); and as an adjunct to histopathology in the surgical setting (18). However, the use of CGM as a non-research, clinical tool has not been evaluated in the inpatient setting.

We aimed to investigate if the use of CGM in the inpatient setting identifies hypoglycemia that would have gone unnoticed on intermittent fingerprick testing. We also sought to measure the accuracy of CGM in the inpatient setting.

## 2 Methods

Since 2023, the use of CGM devices for all inpatients with HI has been standard practice within our center. Between September 2022 and November 2025, all patients admitted to a quaternary specialist HI center, were provided with a Dexcom G6 or G7 CGM device and data were collected for the duration of their inpatient stay. Patients, and their parents, consented to sharing their CGM and finger-prick data as per ethical approval HRA 07/H1010/88. All inpatients with HI, during this period, were included in the study and thus no patients were excluded.

Using Dexcom Clarity, CGM data were reviewed for hypoglycemic events for each patient. Hypoglycemic events were defined as per ADA CGM hypoglycemia criteria; at least three consecutive readings <3.5mmol/L required to “commence” an event and three consecutive readings ≥3.5mmol/L required to terminate the event. All events were thus ≥15 minutes duration. From a database obtained from the biochemistry department of the hospital, paired point of care test (POCT) glucometer finger-prick glucose values were sought for all hypoglycemic events to confirm or refute true hypoglycemia. False positives were those where a fingerprick performed during the hypoglycemic event was ≥3.5mmol/L.

For true positives (fingerprick value <3.5mmol/L during an event), electronic healthcare records were accessed to determine when the next scheduled fingerprick would have taken place, had it not been for the CGM alert. An example CGM trace demonstrating the calculation method is shown in **Figure 1**. The time between the start of the hypoglycemic event and the time of the next scheduled fingerprick was used to determine the length of potential hypoglycemia (red on **Figure 1**). The time between the end of the hypoglycemic event and the next scheduled fingerprick was used to calculate the amount of hypoglycemia likely saved, due to CGM alarms (green on **Figure 1**). These were used to provide context for the relative amount of hypoglycemia saved as a proportion of total time hypoglycemic (green on **Figure 1** as a proportion of red on **Figure 1**). Electronic health records were also reviewed to determine whether the hypoglycemic event had been treated and by what method. Baseline statistics of mean, median, standard deviation, and range were calculated for all hypoglycemia measures, in Microsoft Excel.

**Figure 1:**
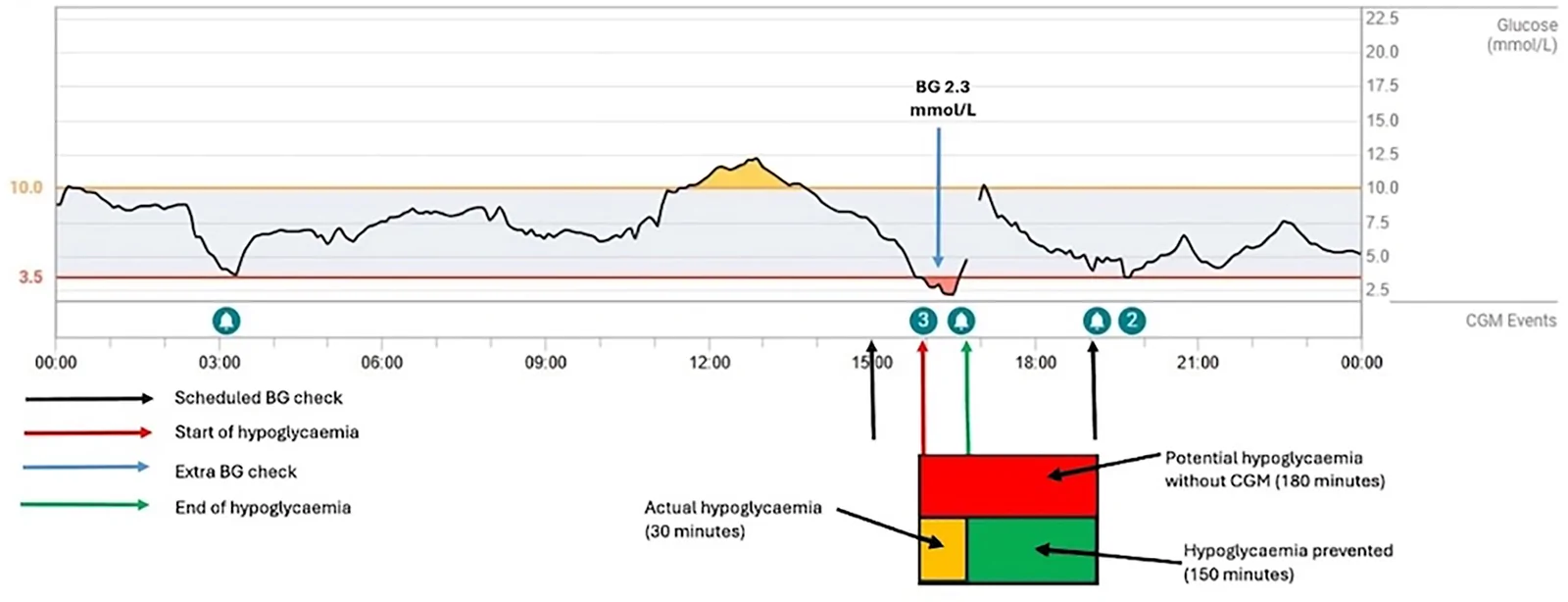
Example of a CGM trace with overlay to explain the method of calculation for “potential hypoglycemia” (red), “actual hypoglycemia” (yellow) and “hypoglycemia saved” (green).

### 2.1 Accuracy and basic glycemic analysis methods

CGM data were downloaded from Dexcom Clarity in.csv file format. Basic glycemic data such as Time Below Range (TBR) was calculated for glucose thresholds of 3.9mmol/L, 3.5mmol/L and 3.0mmol/L using bespoke code in Python 3.10.

Fingerprick glucometer data were obtained for all patients for the dates of their inpatient admission via the biochemistry department of the hospital. Bespoke code in Python 3.10 was used to pair each fingerprick glucose value to the nearest CGM value for each patient, in a process described in detail elsewhere (19). Fingerprick values that did not have a paired CGM value within 5 minutes were discarded from the analysis. Basic statistics such as mean absolute relative difference (MARD), mean absolute difference (MAD), mean difference and hypoglycemia (<3.5mmol/L) sensitivity and specificity were calculated using Python 3.10. Hypoglycemia detection was defined as any CGM glucose value <3.5mmol/L within a 15-minute period before or after a fingerprick value <3.5mmol/L and this rate was calculated using Python 3.10.

Paired values were subsequently plotted on to the hypoglycemia Error Grid (19) to provide clinical insight into CGM deviation within the specific context of HI.

## 3 Results

### 3.1 Main results

Data were available for 13 patients during the monitoring period. Demographics are provided in **Table 1**. This provided 627 days of CGM data; a mean of 48.2 days per patient. Duration of inpatient CGM use ranged from 3 to 136 days per patient. CGM identified 915 hypoglycemic events; 194 events had no associated point of care test (POCT) finger-prick data to confirm or refute hypoglycemia and were therefore discarded. The remainder (721) of events were checked by POCT glucometer, with 273 true hypoglycemic events and 448 false positives. The false discovery rate was thus 0.71 per day. Total measured, and fingerprick confirmed, hypoglycemia was 10312 minutes, with a mean (SD) of 16.44 (8.4) minutes per day. Using methods described above, a calculated total of 10481 minutes of potential hypoglycemia was saved using CGM, with a mean (SD) of 20.5 minutes (13.3) per patient day (**Table 2**). Hypoglycemia saved per patient, as a proportion of potential hypoglycemic time per day, had a mean of 55% and median of 51% and is represented on a per patient basis in **Figure 2**.

**Table 1:** Patient characteristics and treatment strategies.

Patient and age (months)	Genetics	Medical treatment	Max GIR (mg/kg/min)	Diazoxide (mg/kg/day)	Octreotide (mcg/kg/day)	Glucose polymer/feed	Discharge management	Other
*A* *(*1*)*	INSR (dominant)	20% Glucose & Glucagon	20.9	13	20	Polycal 3.5%	Diazoxide & Polycal 3.5%	
*B* *(*174*)*	ABCC8 (recessive)	20% Glucose & Glucagon	20.9	15	20	Vitajoule 2.5%	Focal lesionectomy - Cured	
*C* *(*2*)*	Insulinoma (genetics negative)	10% Glucose	2.4	4		Corn Flour 20 grams	Focal lesionectomy - Cured	
*4* *(*1*)*	ABCC8 (dominant)	15% Glucose & Glucagon	10.3	15	20, 20mg LAR		Pediatric Compleat	Dasiglucagon (10mcg/ 70mcg)
*5* *(*3*)*	ABCC8 (recessive)	50% Glucose & Glucagon	40	15	20		Focal lesionectomy - Cured	
*6* *(*2*)*	ABCC8 (dominant)	20% Glucose & Glucagon	17.2	15	22.5	Vitajoule 2.5%	Octreotide	Blended diet & Nutrini Low Energy Multifiber
*7* *(*4*)*	BWS	20% Glucose & Glucagon	13	12.5	10	Vitajoule 4%	Subtotal pancreatectomy - Diazoxide	
*8* *(*1*)*	ABCC8 (recessive)	20% Glucose & Glucagon	14.4	15	19	Polycal 3.5%	Subtotal pancreatectomy - Octreotide	Octreotide Via Medtronic Pump
*9* *(*6*)*	ABCC8 (recessive)	10% Glucose & Glucagon	10.3	10	8.7	Maxijul 1%	Focal lesionectomy - Cured	
*10* *(*7*)*	ABCC8 (recessive)	20% Glucose & Glucagon	12.3	15	20	Polycal 5%	Focal lesionectomy - Cured	Similac High Energy Formula
*11* *(*7*)*	HNF4A	20% Glucose & Glucagon	11.8	12.5			Diazoxide	
*12* *(*2*)*	ABCC8 (recessive)	20% Glucose & Glucagon	15	15	20		Focal lesionectomy – Dasiglucagon	Dasiglucagon (60mcg)
*13* *(*1*)*	ABCC8 (recessive)	30% Glucose & Glucagon	19.5	15	20	Vitajoule 5%	Subtotal pancreatectomy - Octreotide	Octreotide Via Medtronic Pump

Patient-level characteristics including age, genetic diagnosis and age along with their medical treatment, maximum glucose infusion rate, diazoxide and octreotide doses, and glucose polymer/feed requirements. Discharge management and other interventions are also included.

*BWS, Beckwith-Wiedemann syndrome.

**Table 2:** Actual and theoretical hypoglycemic time and estimated hypoglycemia saved using CGM.

	Actual total hypoglycemic time (mins)	Total hypoglycemic time saved (mins)	Potential total hypoglycemic time (mins)	Actual hypoglycemic time per day (mins)	Potential hypoglycemic time per day (mins)	Total hypoglycemia saved per day (mins)
Total	10312	10481	20793			
Mean	793.2	806.2	1599.5	16.4	36.3	20.5
Median	745	610	1424	14.1	29.8	17.4
Standard Deviation	666.8	638.3	1225.5	8.4	15.8	13.3
Range	1225	690	1915	29.3	50.3	50.8

Summary of total, mean, median, standard deviation, and range for actual and theoretical hypoglycemic time and hypoglycemia saved per day.

**Figure 2:**
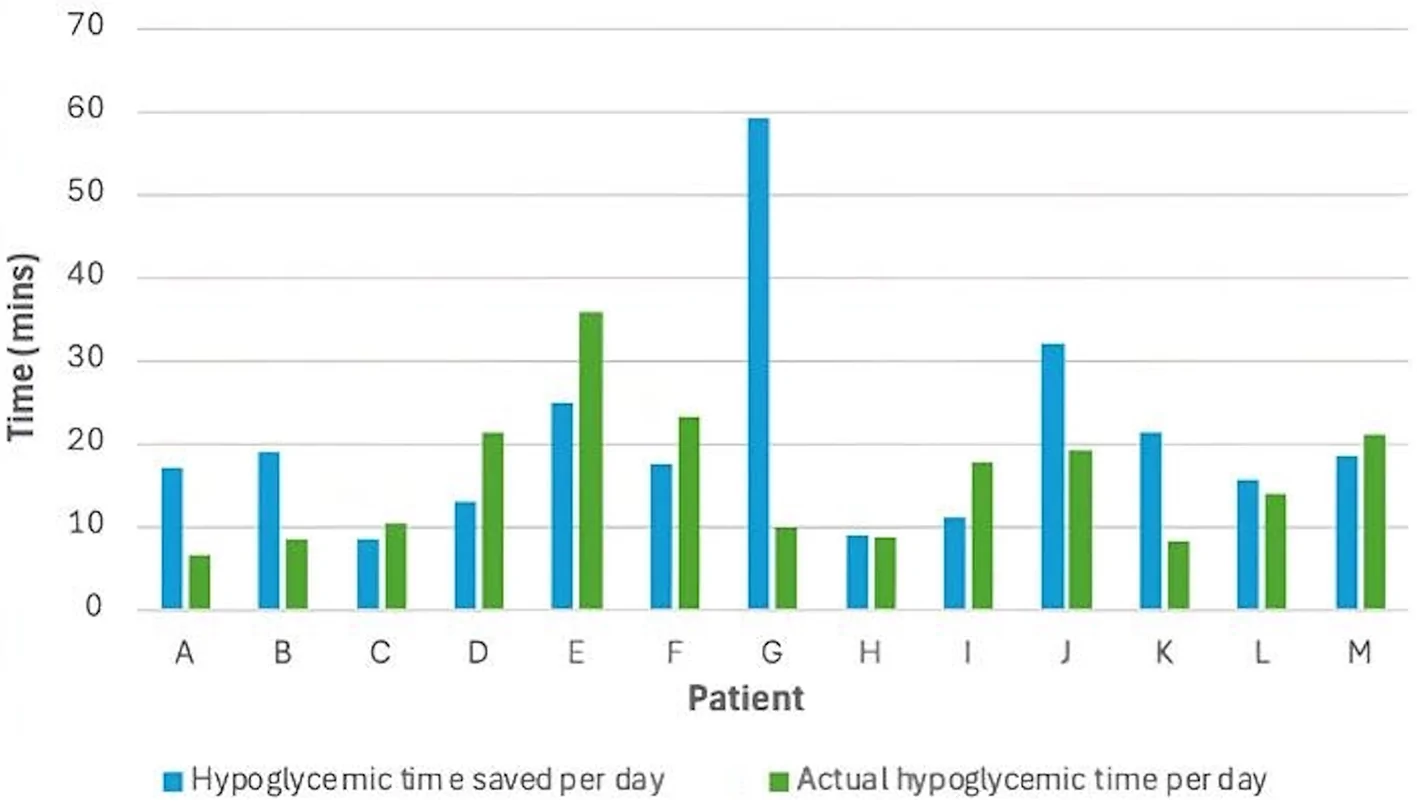
Showing the hypoglycemic time saved per day for each patient and the actual hypoglycemic time per patient in a composite graph. It can be appreciated that Patient G is somewhat of an outlier; this is likely due to the very small number of datapoints for this patient.

There were 146 hypoglycemic events where no finger-prick testing took place during the hypoglycemia. The mean duration of these unconfirmed hypoglycemia events was 32.1 minutes. The lowest CGM glucose reading during these events was 2.7mmol/L.

### 3.2 Accuracy and basic glycemic data results

There were 134, 849 CGM data points available for analysis, representing 11, 237 hours of inpatient glucose monitoring. Mean (range) time below range (<3.5mmol/L) was 4.8% (1.8 - 14.5%). An analysis of hypoglycemia by time of day showed relatively low risk after midnight and before 6am and periods of highest risk in the afternoon (**Figure 3**).

**Figure 3:**
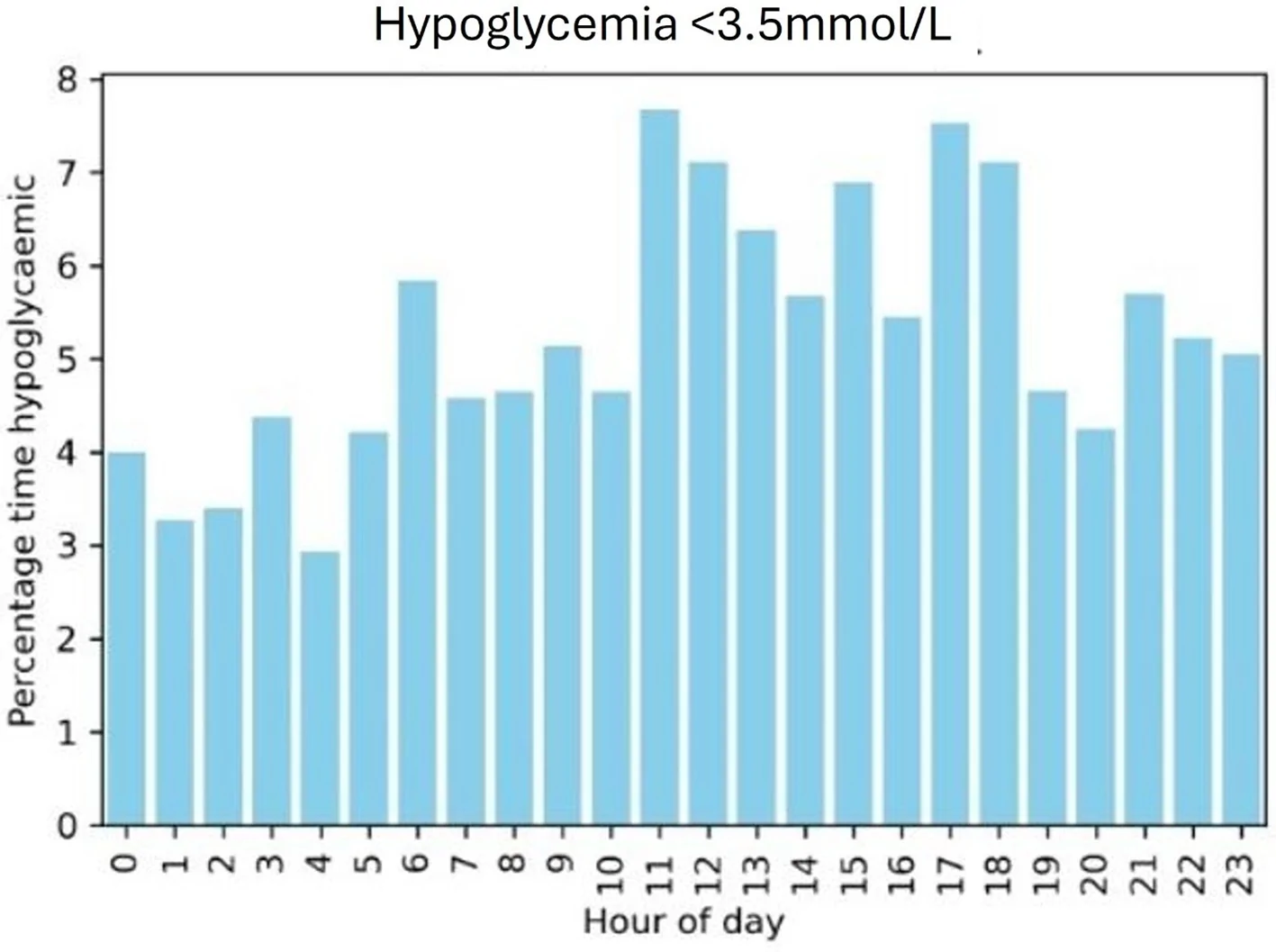
Risk of hypoglycemia by time of day, for all patients. The afternoon appears to be a period of higher risk than overnight or morning.

After the pairing process, there were 3504 paired fingerprick and CGM values for analysis. Mean absolute relative difference (MARD) was 24.1% with CGM devices tending to overread; mean difference was +0.43mmol/L, with absolute difference 1.24mmol/L. Hypoglycemia (<3.5mmol/L) sensitivity was only 38.8% and hypoglycemia detection rate only 56.7%, with 236/416 fingerprick confirmed hypoglycemia events identified by CGM within 15 minutes of fingerprick.

When plotted on the Hypoglycemia Error Grid (HEG), 89.4% of values were within Zone A, representing no risk (**Figure 4**). There were 5.5% and 0.7% of values within Zones C and D, representing moderate and severe risk respectively (**Figure 4**).

**Figure 4:**
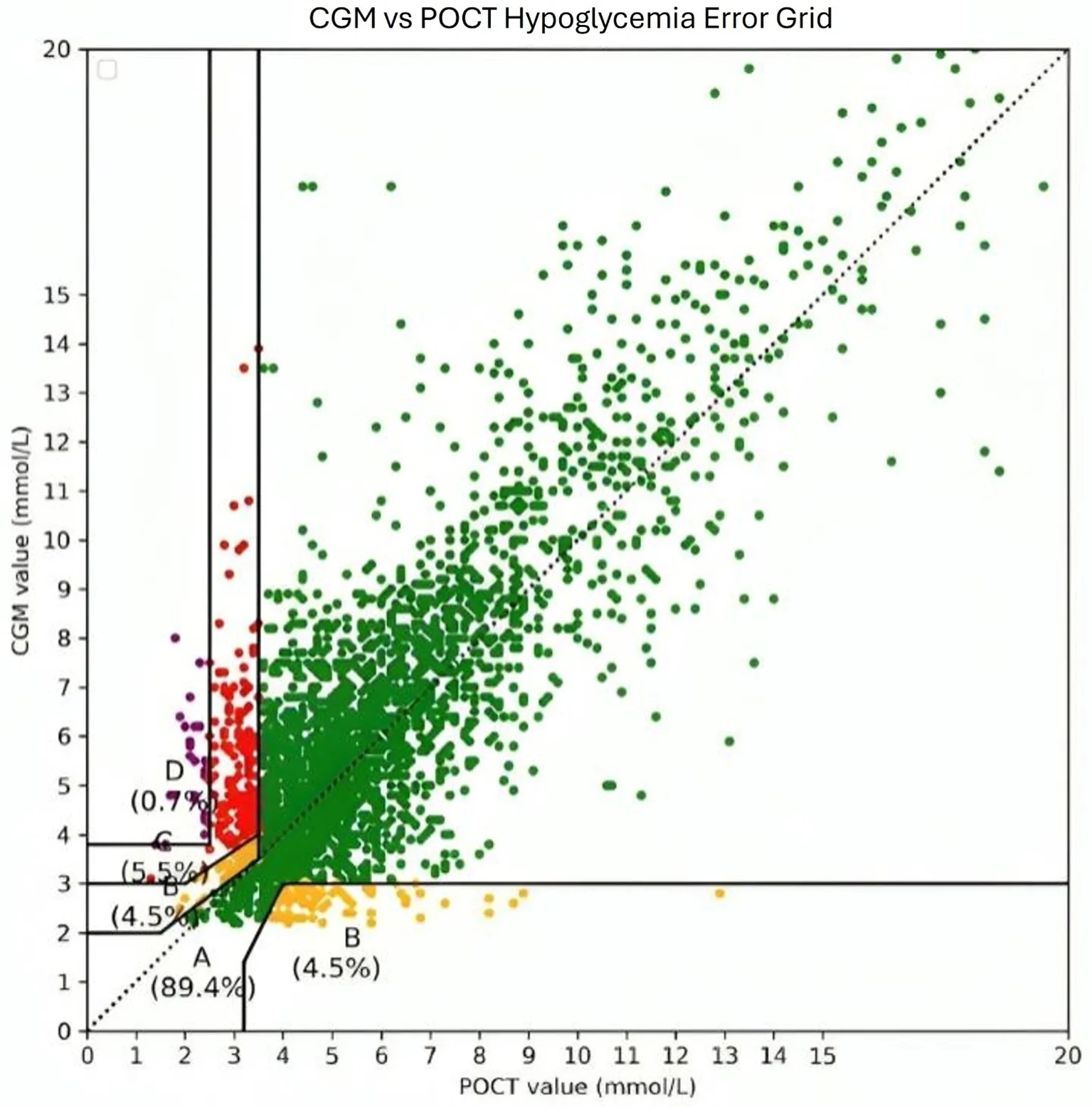
Hypoglycemia Error Grid plotted with paired values. POCT, point of care test (finger-prick value); CGM, continuous glucose monitoring.

### 3.3 Limitations

Several limitations must be recognized. This is a single-center study with a modest sample size—inevitable given the rarity of severe HI. Scheduled fingerprick intervals varied by clinical context, introducing heterogeneity into counterfactual modelling, although the method was applied consistently and reflects real-world practice. It is also possible that planned fingerprick frequency was reduced by the reassuring presence of CGM and this may be acting to falsely increase hypoglycemic time saved. Finally, CGM alarm settings, staff familiarity, and responsiveness to alerts likely influenced hypoglycemia prevention and may limit generalizability to centers less experienced with this technology.

## 4 Discussion

The present study provides the first systematic evaluation of CGM as a routine clinical tool for inpatients with HI. While CGM has been heavily adopted in diabetes care for two decades and has begun to demonstrate perioperative utility in HI (18), its role in the inpatient setting has not been well documented. Our findings show that CGM can materially alter the glycemic landscape of inpatient HI care, identifying hypoglycemia that would have been missed by routine fingerprick monitoring and likely preventing a substantial proportion of otherwise unrecognized events.

Across 627 patient-days of monitoring, CGM identified 915 candidate hypoglycemia events, of which 273 were confirmed biochemically. This represents a sizeable burden of hypoglycemia despite the intensive inpatient care provided at an international center of excellence. This aligns with outpatient findings from wider CGM datasets in HI showing high event frequency (16) and clustering at predictable times of day. Interestingly, clustering in the inpatient setting appears at a different time of day from that seen in multiple studies in the outpatient setting where hypoglycaemia is more commonly seen in the early hours of the morning (16, 17, 20). Further work is warranted, to try and understand this discrepancy. Unlike outpatient studies, however, our inpatient analysis attempts to quantify the counterfactual—that is, how much hypoglycemia would likely have occurred without CGM-triggered intervention. By examining scheduled fingerprick intervals and aligning them to CGM-detected events, we estimate that CGM saved an average of 20.5 minutes of hypoglycemia per patient-day, effectively halving the hypoglycemic burden.

This approach moves beyond previous accuracy studies and measures real-world clinical impact. Previous evaluations—including our own work on the Hypoglycemia Error Grid—have demonstrated that point accuracy and hypoglycemia sensitivity of CGM in HI are consistently suboptimal. The present data do not contradict this; indeed, our paired analysis confirms familiar patterns of over-reading and poor hypoglycemia sensitivity. Yet these limitations did not prevent CGM from having a meaningful protective effect for vulnerable patients. This reinforces a key principle of using CGM for those with HI: CGM provides greatest value when it is used as a trend-monitoring and behavior-modifying tool, rather than as a surrogate for plasma glucose monitoring via finger-prick.

While the proportion of finger-prick refuted CGM hypoglycemia events (448/721) is high, and reflects known accuracy limitations, the burden is lower in the inpatient setting. In the inpatient environment alarms served as alerts to risk, often prompting earlier bedside glucose checks, increased vigilance, or anticipatory adjustments in intravenous therapy. These alarms are often interpreted by medical professionals, and the often-high burden of alarm fatigue is not felt solely by caregivers. The clinical cost of false positives is therefore lower in this setting, whereas the potential benefit of preventing prolonged or severe hypoglycemia remains high.

Notwithstanding the documented limitations, this study provides the clearest evidence to date that CGM can reduce hypoglycemia exposure for children with HI during hospital admission. We suggest that CGM should be considered for all inpatients with severe HI, particularly those with unpredictable glycemic profiles or requiring high-frequency monitoring. Larger multicenter studies—ideally using standardized sampling protocols, are warranted to evaluate generalizability and to refine estimates of hypoglycemia prevention. This study utilised data from 13 inpatients all of whom had varying inpatient lengths. For example, Patient G contributed very few data points (4 finger-pricks recorded) due to their short inpatient stay. While they appear to be an outlier, we chose to leave this data in our study as the small number of data points would not unduly influence mean effects of the overall outcome of the study.

More broadly, CGM in HI must be understood as a tool for trend-driven decision support rather than numeric glucose replacement. As HI care increasingly incorporates digital phenotyping and algorithmic support, the role of CGM will expand beyond measurement to active risk management.

## Data Availability

The raw data supporting the conclusions of this article will be made available by the authors, without undue reservation.
